# Relationships between Sphaerulina musiva Infection and the *Populus* Microbiome and Metabolome

**DOI:** 10.1128/msystems.00120-22

**Published:** 2022-07-18

**Authors:** Nicholas C. Dove, Alyssa A. Carrell, Nancy L. Engle, Dawn M. Klingeman, Miguel Rodriguez, Toni Wahl, Timothy J. Tschaplinski, Wellington Muchero, Christopher W. Schadt, Melissa A. Cregger

**Affiliations:** a Biosciences Division, Oak Ridge National Laboratory, Oak Ridge, Tennessee, USA; b Department of Microbiology, University of Tennessee, Knoxville, Tennessee, USA; c Department of Ecology & Evolutionary Biology, University of Tennessee, Knoxville, Tennessee, USA; Lawrence Berkeley National Laboratory

**Keywords:** 16S rRNA, ITS, metabolomics, microbial ecology, plant endosphere, plant-microbe interactions, rhizosphere, *Septoria*, phyllosphere

## Abstract

Pathogenic fungal infections in plants may, in some cases, lead to downstream systematic impacts on the plant metabolome and microbiome that may either alleviate or exacerbate the effects of the fungal pathogen. While Sphaerulina musiva is a well-characterized fungal pathogen which infects *Populus* tree species, an important wood fiber and biofuel feedstock, little is known about its systematic effects on the metabolome and microbiome of *Populus*. Here, we investigated the metabolome of Populus trichocarpa and Populus deltoides leaves and roots and the microbiome of the leaf and root endospheres, phylloplane, and rhizosphere to understand the systematic impacts of *S. musiva* abundance and infection on *Populus* species in a common garden field setting. We found that *S. musiva* is indeed present in both *P. deltoides* and *P. trichocarpa*, but *S. musiva* abundance was not statistically related to stem canker onset. We also found that the leaf and root metabolomes significantly differ between the two *Populus* species and that certain leaf metabolites, particularly the phenolic glycosides salirepin and salireposide, are diminished in canker-infected *P. trichocarpa* trees compared to their uninfected counterparts. Furthermore, we found significant associations between the metabolome, *S. musiva* abundance, and microbiome composition and α-diversity, particularly in *P. trichocarpa* leaves. Our results show that *S. musiva* colonizes both resistant and susceptible hosts and that the effects of *S. musiva* on susceptible trees are not confined to the site of canker infection.

**IMPORTANCE** Poplar (*Populus* spp.) trees are ecologically and economically important trees throughout North America. However, many western North American poplar plantations are at risk due to the introduction of the nonnative fungal pathogen *Sphaerulina musiva*, which causes leaf spot and cankers, limiting their production. To better understand the interactions among the pathogen *S. musiva*, the poplar metabolome, and the poplar microbiome, we collected leaf, root, and rhizosphere samples from poplar trees consisting of 10 genotypes and two species with differential resistance to *S. musiva* in a common garden experiment. Here, we outline the nuanced relationships between the poplar metabolome, microbiome, and *S. musiva*, showing that *S. musiva* may affect poplar trees in tissues distal to the site of infection (i.e., stem). Our research contributes to improving the fundamental understanding of *S. musiva* and *Populus* sp. ecology and the utility of a holobiont approach in understanding plant disease.

## INTRODUCTION

Poplar (*Populus* spp.) trees are an important biofuel feedstock (1), commercial fiber source (2), and foundational species in many ecosystems throughout North America (3). However, many western North American poplar plantations and ecosystems may be at risk due to the introduction of the fungal pathogen Sphaerulina musiva, which is native only to Eastern North America (synonym, Septoria musiva; teleomorph, Mycosphaerella populorum) (4, 5). In susceptible native species and hybrids, *S. musiva* causes early leaf drop and development of stem cankers, potentially limiting the production of poplar products and damaging riparian ecosystems (5–7). When severe, leaf spot causes premature leaf defoliation, and infections can spread to the stem, causing cankers, which girdle stems, often causing breakage of the primary stem and branches, eventually killing the tree (6, 8). Recent research has begun to consider pathogenesis from a holobiont perspective, moving toward integrating knowledge of the interactions among plant genetics, the plant metabolome, and the plant microbiome to understand effects of pathogens across the host (9–11). These interactions, in part, may moderate the susceptibility of numerous plants to fungal, bacterial, and viral infections. However, even though poplar trees have emerged as a model species for plant-microbe interactions (12, 13), the impact of *S. musiva* on the overall poplar metabolome and microbiome, including tissues distal to the site of infection (i.e., the stem), is thus far unknown.

Interestingly, different poplar species and genotypes show different resistance to *S. musiva*. For instance, Populus deltoides, endemic to the southeastern United States and Great Plains regions, evolved in the presence of *S. musiva* (4, 14). While *S. musiva* can cause leaf spot and drop in *P. deltoides*, these trees are able to resist *S. musiva* canker infection and mortality (15). Alternatively, Populus trichocarpa, which is native to northwestern North America, has only recently been introduced to *S. musiva*, resulting in documented deleterious effects on poplar plantations (5) with the potential to cause damage to native poplars and riparian ecosystems (7). While *P. trichocarpa* and *P. trichocarpa* × *deltoides* hybrids are generally susceptible to *S. musiva* infection (7), different *P. trichocarpa* genotypes show variable resistance to *S. musiva* infection (16). In a genome-wide association study (GWAS) of a *P. trichocarpa* population challenged with *S. musiva*, resistant genotypes were correlated with genes encoding a putative membrane-bound L-type receptor-like kinase and two receptor-like proteins, while susceptible genotypes were associated with a G-90 receptor-like kinase (16). These receptors are thought to potentially impact the ability of these genotypes to recognize *S. musiva* early and initiate immune responses (16). However, plant immune responses may also be moderated by plant metabolomes and microbiomes both at the site of infection and throughout the plant (17, 18), and as a result, it is often unclear how greenhouse-based assays of resistance will translate to field settings due to these and other environmental interactions. Thus, incorporating the effects of *S. musiva* among different species and genotypes with different resistances could allow a more comprehensive understanding of this pathogen and its ability to infect poplar trees. Furthermore, such observations in field-based studies that are carried out over longer growth periods could potentially provide more practical information on *S. musiva* infection of poplars in natural and plantation settings.

Metabolomic profiles between *P. trichocarpa* and *P. deltoides* show that important tradeoffs exist among pathogen defense, environmental stress, mycorrhizal colonization, and growth (19–21). Hence, the metabolomic response to pathogen infection is likely a key mediator of pathogen resistance. For instance, inoculation of the ectomycorrhizal fungus Laccaria bicolor into different poplar species results in increased metabolites that facilitate colonization in *P. trichocarpa* and has been shown to increase defense compounds that deter colonization in *P. deltoides* (19). It is therefore possible that these and similar compounds might mitigate the severity of *S. musiva* or other fungal infections in *P. deltoides*. Within *P. trichocarpa*, resistant and susceptible genotypes differentially produced numerous metabolites, including signaling molecules, organic acids, amino acids, sterols, phenolics, and saccharides, upon inoculation with *S. musiva* (18). Taken together, these findings suggest that metabolomic differences between and within poplar species may have the potential to modulate the impacts of *S. musiva* on poplar trees.

Similarly, plant microbiomes may also be key mediators and indicators of pathogen resistance in poplar trees (22). For instance, certain fungal endophytes have been shown to moderate or facilitate the effects of the fungal pathogens *Melampsora* × *columbiana* (23) and Drepanopeziza populi (24) in a variety of poplar species. Furthermore, previous attempts have been made to mitigate *S. musiva* leaf spot using Phaeotheca dimorphospora (25) and multiple *Streptomyces* strains (26) with mixed success. Recently, Bacillus velezensis EB14, an endophytic bacterial strain, was shown to have antagonistic effects toward *S. musiva* stem canker infections (27). Regardless, it is still unclear how the characteristics of the overall microbiome of poplar trees are affected by, and may moderate, *S. musiva* infection.

While the plant metabolome and microbiome may modify pathogen infection, it is also important to recognize that such impacts may be bidirectional. In other words, pathogen infection can affect the plant metabolome, resulting in changes to the microbiome, and/or may affect the microbiome directly (17). Such impacts may result in additional indirect consequences for plant health, as the microbiome also influences nutrient use, growth rate and allocation, and stress tolerance (11). Therefore, a complete, systems-based understanding of the impact of plant pathogens should include interactions among infecting pathogens and the host, as well as the microbiome and metabolome. Such comprehensive approaches to systems-level understandings have been termed holobiont research (9, 12, 28).

To better understand the interactions among the pathogen *S. musiva*, the *Populus* metabolome, and microbiome of the *Populus* holobiont, we collected leaf, root, and rhizosphere samples from 75 *Populus* trees consisting of 10 genotypes and two species within a common garden experiment in the eastern United States, where *S. musiva* is prevalent (16). We scored these trees for *S. musiva* canker infection to assess associations between canker infection and the aforementioned data sets. *S. musiva* presence was confirmed via cultures isolated from stem and branch cankers. In addition to culture characterization, isolates were verified by sequencing to confirm identities. Stem cankers caused by this pathogen are characteristically moist and collapsed, eventually resulting in stem and branch breakage. These symptoms are distinct from those of other cankers caused by insects or other pathogens. Unfortunately, we were unable to score the trees for leaf spot, which can also be caused by *S. musiva* (among other pathogens), so “site of infection” in this paper refers only to the stem.

We hypothesized that *S. musiva* abundance throughout the tree would correlate with stem canker infection, particularly in susceptible genotypes. We also hypothesized that *S. musiva* abundance and stem canker infection would impact the metabolome, specifically an increase in phenolic glycosides, which are released by plants for pathogen defense (29). Finally, we hypothesized that *S. musiva* abundance and stem canker infection would impact the microbiome throughout the tree both directly and indirectly, through impacts on the plant metabolome. Our overall goal was to integrate observations across data sets to better understand interactions among *S. musiva*, the *Populus* metabolome, and the *Populus* microbiome. Such information could be used to improve the sustainability and productivity of *Populus* and other tree species in the face of increasing pathogen introductions and disease pressure in managed and natural ecosystems.

## RESULTS

### *S. musiva* abundance among plant-associated habitats is unrelated to stem canker infection.

**FIG 1:**
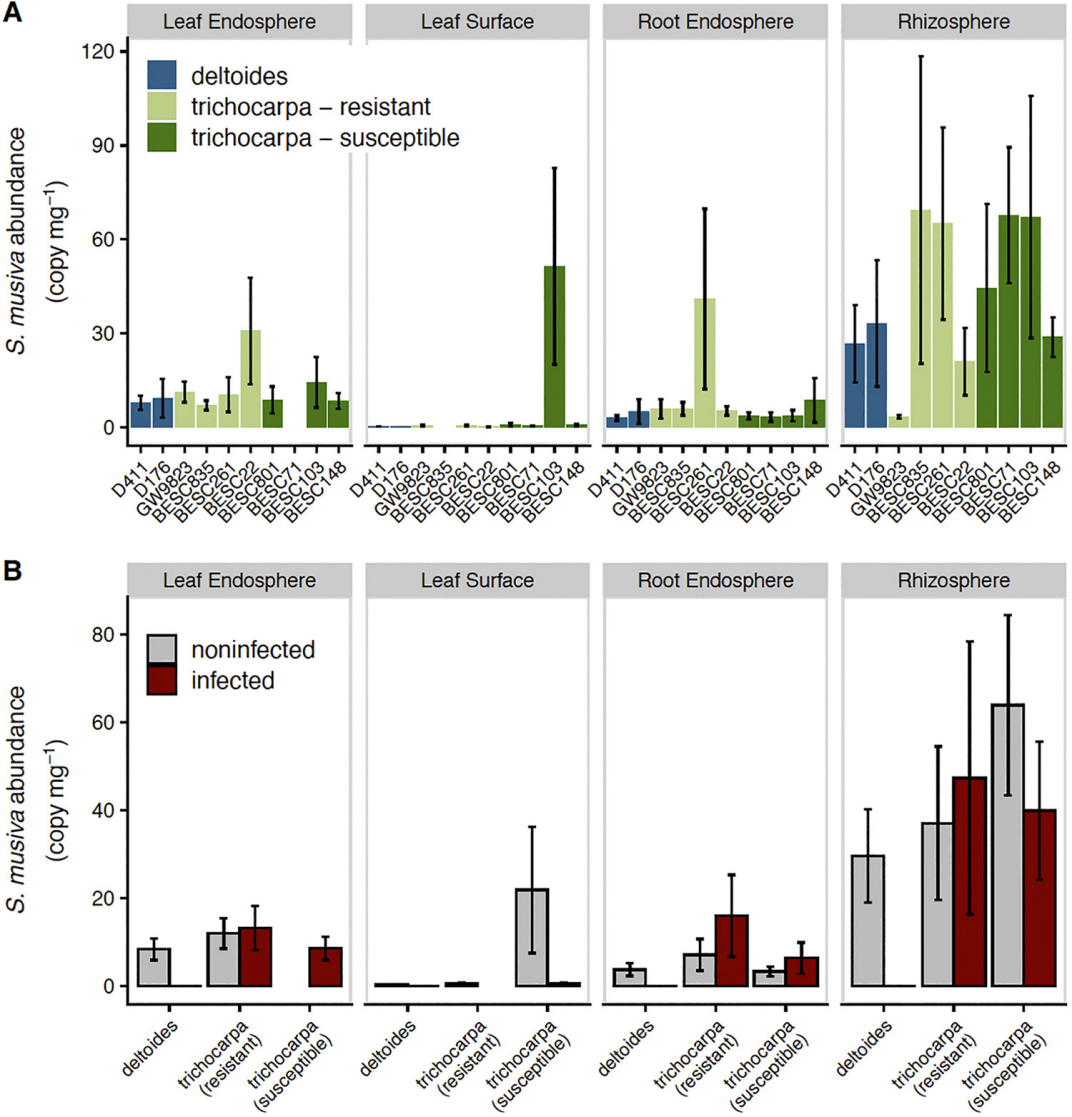
Mean (and standard error) *Sphaerulina musiva* abundance among genotypes (A) and levels of infection (B) across sample types. *Populus trichocarpa* resistance to *S. musiva* is classified as in reference 16.

*S. musiva* was present in qPCR assays across all plant-associated habitats in all *Populus* species and most genotypes, with the greatest abundance of *S. musiva* in the rhizosphere (analysis of variance [ANOVA]: *F*_3,253_ = 60.6, *P < *0.001) (Fig. 1A). However, *S. musiva* abundance in different plant-associated habitats was uncorrelated (all correlations: *P > *0.05) and was unaffected by genotype (*F*_9,253_ = 0.9, *P = *0.522), host species (*F*_1,253_ = 0.03, *P = *0.873), or its reported resistance or susceptibility in *P. trichocarpa* (*F*_2,142_ = 0.09, *P = *0.764) (Fig. 1A). Canker infection was absent in *P. deltoides*, and *S. musiva* abundance was not indicative of canker infection for *P. trichocarpa* (binomial regression, all plant-associated habitats: *P > *0.05) (Fig. 1B). Furthermore, the previously reported resistance or susceptibility of *P. trichocarpa* genotypes from 21-day greenhouse assays (16) did not affect the relationship between canker infection and *S. musiva* abundance in this field setting (*P > *0.05). *S. musiva* abundance, as measured by qPCR, did not correlate with the relative abundance of *Sphaerulina* in the internal transcribed spacer (ITS) data set (taxonomic assessment below the genus level was undefined; Spearman rho: *P = *0.720, *n* = 133). This is likely due to biases in ITS amplification (30), incomplete reference databases (31), or challenges in relating relative and total abundances (32).

### The *P. trichocarpa* leaf metabolome is moderately affected by *S. musiva* canker infections.

Host species explained 33% and 23% of the variation in the leaf and root metabolome, respectively (permutational multivariate ANOVA [PERMANOVA]: leaf, *R*^2^ = 0.33, *P < *0.001; root, *R*^2^ = 0.23, *P < *0.001) (see Fig. S1 in the supplemental material). Among *P. trichocarpa* genotypes, there was also a significant genotype effect on the leaf and root metabolome (leaf, *R*^2^ = 0.21, *P < *0.001; root, *R*^2^ = 0.21, *P < *0.001). This was apparent as a weak effect of previously reported *S. musiva* resistance or susceptibility on the root metabolome (*R*^2^ = 0.04, *P = *0.030) but not the leaf metabolome (*P > *0.05).

10.1128/msystems.00120-22.3 FIG S1 Principal-component (PC) analysis of leaf (A) and root (B) metabolites. The percentage in parentheses represents the variance explained by each axis (i.e., component). Note different axis scales. Download FIG S1, JPG file, 0.6 MB. Copyright © 2022 Dove et al. 2022 Dove et al. https://creativecommons.org/licenses/by/4.0/ This is an open-access article distributed under the terms of the Creative Commons Attribution 4.0 International license.

**FIG 2:**
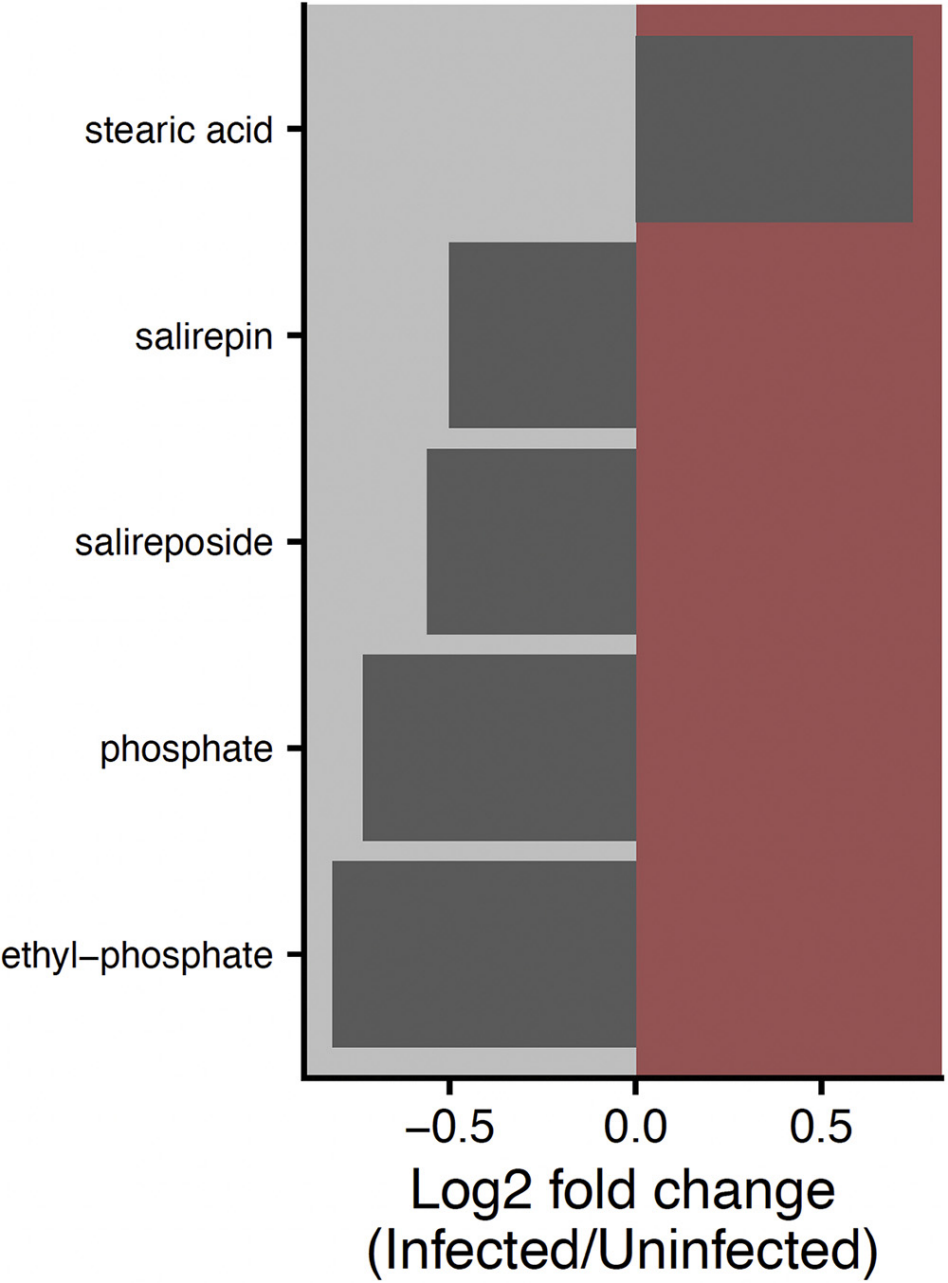
Significant (*P*_adj_ < 0.05) log_2_ fold differences in leaf metabolites between infected (red) and noninfected (gray) *Populus trichocarpa* trees.

Canker infection in *P. trichocarpa* was associated with a small but significant effect on the whole-leaf metabolome (PERMANOVA: *R*^2^ = 0.03, *P = *0.024; Fig. S1), namely, five individual metabolites that were differentially abundant (Wilcoxon rank sum test: adjusted *P* [*P*_adj_] < 0.05) (Fig. 2). Stearic acid was found to be 67% higher in infected *P. trichocarpa* tree leaves, the only metabolite that increased with infection (Fig. 2). The four other differentially abundant metabolites included the phosphorus-containing compound ethyl-phosphate and phosphate itself, which were 75% and 66% more abundant in uninfected tree leaves, respectively, as well as the phenolic glycosides salirepin and salireposide, which were 41% and 47% more abundant in uninfected tree leaves, respectively (Fig. 2). These phenolic glycosides were also differentially abundant between *P. deltoides* and resistant *P. trichocarpa*, with salirepin being significantly enriched in *P. deltoides* and salireposide enriched in resistant *P. trichocarpa* (*P*_adj_ < 0.05) (Fig. S2). The root metabolome, on the other hand, was not found to be associated with canker infection (PERMANOVA: *P = *0.504) (Fig. S1). In both the leaf and the root, no individual metabolites were significantly correlated with *S. musiva* abundance assessed by qPCR after adjusted for multiple comparisons (Spearman correlation: *P*_adj_ > 0.05).

10.1128/msystems.00120-22.4 FIG S2 Box plots representing differences in salirepin (A) and salireposide (B) concentrations between *P. deltoides* and resistant *P. trichocarpa*. Download FIG S2, JPG file, 0.3 MB. Copyright © 2022 Dove et al. 2022 Dove et al. https://creativecommons.org/licenses/by/4.0/ This is an open-access article distributed under the terms of the Creative Commons Attribution 4.0 International license.

### Microbiome α-diversity differs between species and is associated with *S. musiva* abundance and plant metabolites.

Differences in microbial α-diversity were compared by means of Hill numbers (33) at orders of *q* of 0, 1, and 2. The parameter *q* determines the relative weighting of rare species. At a *q* of 0, all species are weighted equally (richness); at a *q* of 1, species are weighted proportionally to their relative abundance (analogous to Shannon’s index); and at a *q* of 2, rare species are down-weighted (analogous to Simpson’s index).

Alpha diversity varied between *Populus* species, but this was dependent on the plant-associated habitat, microbial domain, and order of *q*. For example, leaf endosphere archaeal and bacterial α-diversity was consistently significantly greater in *P. deltoides* than *P. trichocarpa* across orders of *q* (Wilcoxon rank sum test: *P < *0.05; Table S1). However, root endosphere archaeal and bacterial α-diversity was greater in *P. trichocarpa* than *P. deltoides* only at *q* if 0 and 1, implying that this effect was mainly driven by rare taxa (Table S1). For fungi, the leaf surface had a greater α-diversity in *P. trichocarpa* than *P. deltoides*, but only at a *q* of 0, while the rhizosphere had a greater α-diversity in *P. deltoides*, but only at *q* of 1 and *q* 2 (Table S1). However, across plant-associated habitats, microbial domains, and orders of *q*, α-diversity was not associated with the previously reported resistance or susceptibility of *P. trichocarpa* genotypes or canker infection score in *P. trichocarpa* (*P > *0.05) (Table S1).

10.1128/msystems.00120-22.1 TABLE S1 *P* values for Wilcoxon rank sum tests for differences in α-diversity between host species (Species), previously reported resistance or susceptibility to *Sphaerulina musiva* infection (Resistance), and canker infection (Infection) across different amplicons, sample types, and Hill number *q* factors (*q* = 0 is analogous to richness, *q* = 1 is analogous to Shannon diversity, and *q* = 2 is analogous to Simpson diversity). Bold *P* values represent significant differences (*P < *0.05). Download Table S1, DOCX file, 0.01 MB. Copyright © 2022 Dove et al. 2022 Dove et al. https://creativecommons.org/licenses/by/4.0/ This is an open-access article distributed under the terms of the Creative Commons Attribution 4.0 International license.

**FIG 3:**
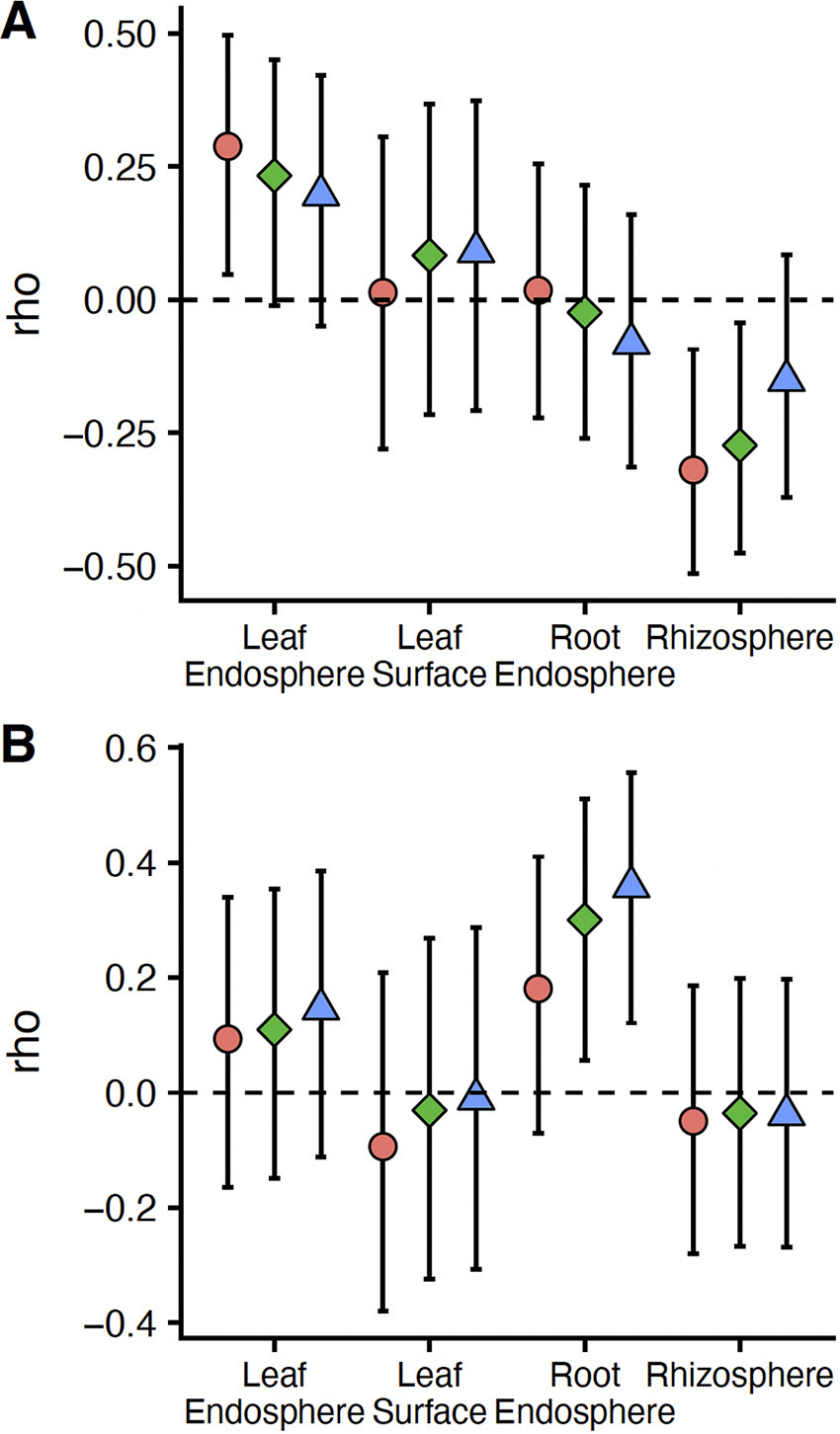
Spearman rho (and 95% confidence intervals) of correlations between *Sphaerulina musiva* abundance and α-diversity in Hill numbers (*q* = 0 is analogous to richness, *q* = 1 is analogous to Shannon diversity, and *q* = 2 is analogous to Simpson diversity) across plant-associated habitats for *Archaea* and *Bacteria* (A) and *Fungi* (B).

Alpha diversity was positively and negatively associated with *S. musiva* abundance in qPCR assays depending on the plant-associated habitat, microbial domain, and order of *q*. For example, at a *q* of 0, archaeal and bacterial α-diversity was positively and negatively associated with *S. musiva* abundance in the leaf endosphere and rhizosphere, respectively (leaf, *P* = 0.020, rho = 0.29; rhizosphere, *P = *0.007, rho = −0.32) (Fig. 3). However, while archaeal and bacterial α-diversity at a *q* of 1 also decreased with increasing *S. musiva* abundance in the rhizosphere, no other archaeal and bacterial α-diversity metric significantly correlated with *S. musiva* abundance in the leaf endosphere (*P > *0.05) (Fig. 3). For fungi, root endosphere α-diversity at *q* values of 1 and 2 was positively associated with *S. musiva* abundance (*q* = 1: *P = *0.017, rho = 0.030; *q* = 2: *P = *0.004, rho = 0.35) (Fig. 3), while α-diversity in other plant-associated habitats and at different orders of *q* was unrelated to *S. musiva* abundance (*P > *0.05) (Fig. 3).

**FIG 4:**
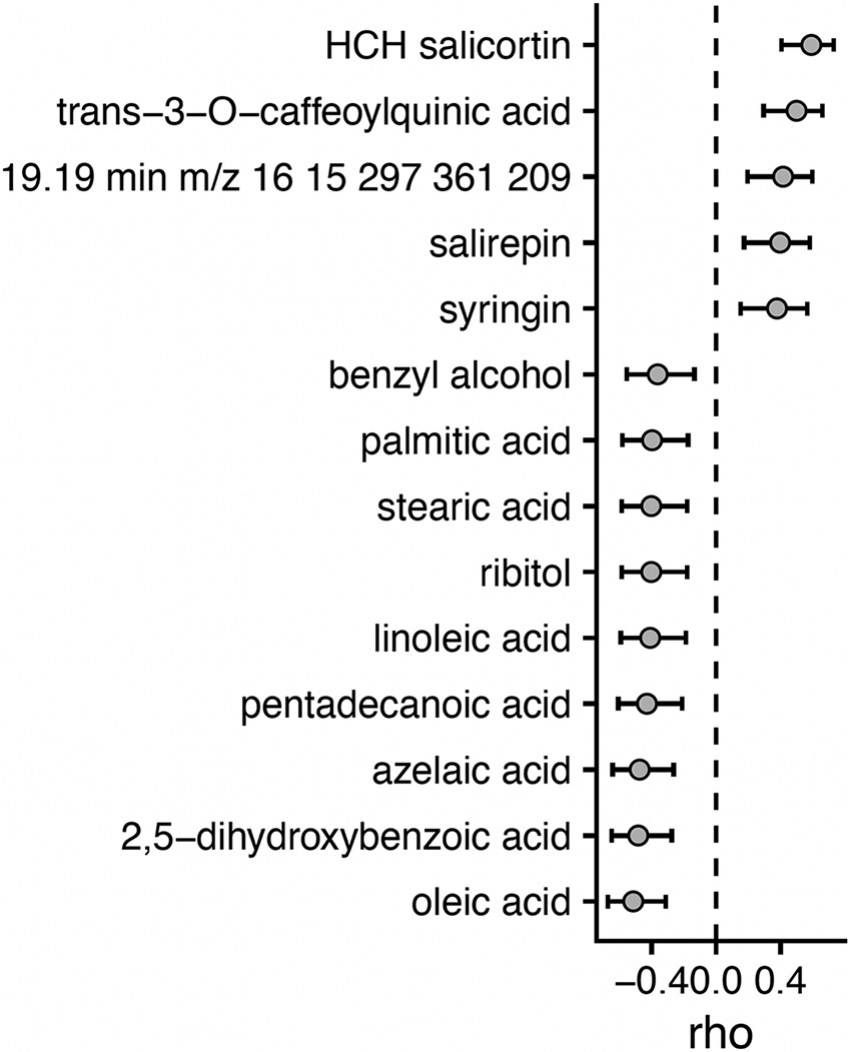
Spearman rho (and 95% confidence intervals) of correlations between *Archaea* and *Bacteria* α-diversity (*q* = 0, i.e., richness) in the leaf endosphere and leaf metabolite concentrations. Only significant (*P*_adj_ < 0.05) values are shown.

Fourteen metabolites were significantly correlated with archaeal and bacterial α-diversity at a *q* value of 0 in the leaf endosphere (Spearman correlation: *P*_adj_ < 0.05) (Fig. 4). Three of these metabolites, HCH-salicortin, oleic acid, and trans-3-*O*-caffeoylquinic acid, were also significantly correlated with archaeal and bacterial α-diversity at *q* = 1 and 2 in the leaf endosphere. Stearic acid, which was higher in leaves of canker-infected *P. trichocarpa* trees (Fig. 2), was negatively correlated with archaeal and bacterial α-diversity at a *q* of 0 in the leaf endosphere, and salirepin, which was lower in leaves of canker-infected *P. trichocarpa* trees (Fig. 2), was positively correlated with archaeal and bacterial α-diversity at a *q* of 0 in the leaf endosphere (Fig. 4). No metabolites were significantly correlated with fungal α-diversity across orders of *q* in the leaf endosphere (*P*_adj_ > 0.05), and no root metabolites were significantly associated with any measures of α-diversity across microbial domains (*P*_adj_ > 0.05).

### Microbiome composition differs between species and is associated with *S. musiva* abundance and plant metabolites.

The compositions of *Archaea* and *Bacteria* and of *Fungi* differed between host species in all plant-associated habitats besides the root endosphere (Table 1). Within *P. trichocarpa*, there was also a significant effect of genotype on the composition of the archaeal and bacterial leaf surface, root endosphere, and rhizosphere microbiomes as well as the fungal leaf endosphere, leaf surface, and rhizosphere microbiomes (Table 1). However, for the most part, this was not related to *S. musiva* resistance/susceptibility, with significant differences in composition between resistant and susceptible *P. trichocarpa* genotypes only in the fungal leaf endosphere (Table 1).

**TABLE 1:** PERMANOVA results of the effects of various characteristics on community composition across amplicons and plant-associated habitats^*a*^

Amplicon	Characteristic	Leaf endosphere		Leaf surface		Root endosphere		Rhizosphere	
		*P*	*R* ^2^	*P*	*R* ^2^	*P*	*R* ^2^	*P*	*R* ^2^
16S	Species	**<0.001**	**0.12**	**<0.001**	**0.10**	0.116	0.02	**0.027**	**0.02**
	Genotype	0.330	0.13	**0.009**	**0.21**	**0.010**	**0.15**	**0.010**	**0.15**
	Resistance	0.538	0.01	0.066	0.03	0.309	0.02	0.328	0.02
	Infection	0.349	0.02	0.516	0.02	0.319	0.02	0.056	0.03

ITS	Species	**<0.001**	**0.12**	**0.002**	**0.09**	0.198	0.02	**0.039**	**0.04**
	Genotype	**<0.001**	**0.33**	**0.044**	**0.20**	0.631	0.12	**0.003**	**0.18**
	Resistance	**<0.001**	**0.11**	0.053	0.04	0.198	0.02	0.080	0.03
	Infection	0.935	0.01	0.559	0.02	0.513	0.02	0.155	0.02

a The PERMANOVA for the effects of genotype, resistance to *Sphaerulina musiva* infection, and canker infection included only *Populus trichocarpa* trees. Boldface indicates significant moderators (*P < *0.05).

**FIG 5:**
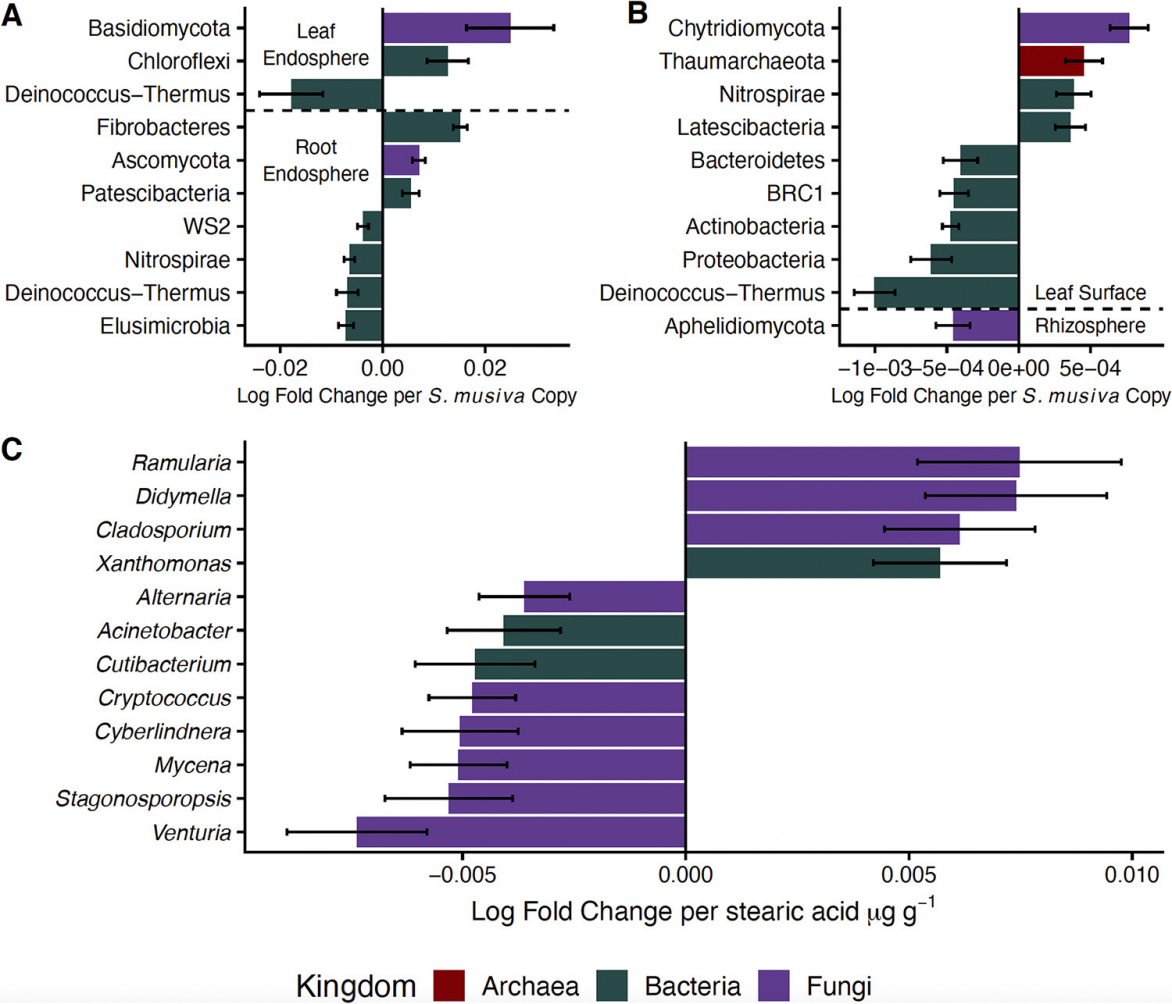
Coefficients (and standard errors) of significant correlations (*P*_adj_ < 0.05) between the relative abundance of microbial phyla and *S. musiva* abundance across plant endosphere habitats (A) and plant surface habitats (B). Also shown is the relative abundance of microbial genera and stearic acid in the leaf endosphere of *P. trichocarpa* (C) determined by ANCOM-BC.

Across plant-associated habitats, the overall microbiome composition between infected and noninfected *P. trichocarpa* trees was consistent (Table 1), and generally, *S. musiva* abundance did not correlate with the overall microbiome composition. The exception was a small but significant effect in the archaeal and bacterial root endosphere (*R*^2^ = 0.02, *P = *0.046; all others, *P > *0.05). However, *S. musiva* abundance was significantly related to the relative abundance of individual microbial phyla across plant-associated habitats (Fig. 5). For instance, increased *S. musiva* abundance was associated with a greater dominance of *Basidiomycota* and *Chloroflexi* in the leaf endosphere (Fig. 5A). On the leaf surface, there was a shift from the dominance of *Bacteroidetes* and *Proteobacteria* to *Nitrospirae* and *Thaumarchaeota* with increasing *S. musiva* abundance (Fig. 5B). Interestingly, in the root endosphere, *S. musiva* abundance was negatively correlated with *Nitrospirae* dominance and was instead associated with increased dominance of “*Candidatus* Patescibacteria” and Ascomycota (Fig. 5A).

The leaf endosphere microbiome composition was significantly associated with the leaf metabolome (Mantel test: *Archaea* and *Bacteria*, *P = *0.031, rho = 0.14; *Fungi*, *P < *0.001, rho = 0.24), although similar correlations in the root were not significant (*P > *0.05). This is likely due to the strong differences in the leaf metabolome across species (Fig. S1), because upon analysis by host species separately, these correlations were no longer significant (*P > *0.05). However, the five leaf metabolites that were differentially abundant between infected and noninfected *P. trichocarpa* trees (Fig. 2) were associated with certain microbial genera in the *P. trichocarpa* leaf endosphere microbiome, showing that even within a host species, leaf metabolites may contribute to structuring the leaf microbiome. For instance, stearic acid, which was more abundant in the leaves of infected *P. trichocarpa* trees (Fig. 2), was associated with increased dominance of *Cladosporium*, *Didymella*, and *Cutibacterium* at the expense of *Alternaria*, *Venturia*, and *Xanthomonas* (analysis of compositions of microbiomes with bias correction [ANCOM-BC]: *P*_adj_ < 0.05) (Fig. 5C). Alternatively, *Venturia* was positively associated with the phenolic glycoside salireposide (log fold change per μg salireposide g^−1^ = 8.1 × 10^−4^; *P*_adj_ = 0.003), which was greater in noninfected *P. trichocarpa* trees. Similarly, *Cyberlindnera* was positively associated with salirepin, and *Sphingomonas* was positively associated with ethyl phosphate and phosphate (*Cyberlindnera*, log fold change per μg salirepin g^−1^ = 0.011, *P*_adj_ = 0.049; *Sphingomonas*: log fold change per μg ethyl-phosphate g^−1^ = 0.0012, *P*_adj_ = 0.014). Interestingly, both metabolites had higher concentrations in noninfected *P. trichocarpa* trees than in their infected counterparts (Fig. 2).

## DISCUSSION

As *S. musiva* has been recently found in the Pacific Northwest (5), where it is negatively impacting *P. trichocarpa* and hybrid plantations and is poised to spread to native ecosystems, a complete, systems-based understanding of the impact of *S. musiva* on the *Populus* holobiont could be critical to maintain the sustainability of this ecologically and economically important tree species (1, 34). While previous studies have focused on the effect of *S. musiva* at the site of infection (i.e., stem cankers) (18), we show that *S. musiva* is present throughout the tree (Fig. 1) and is associated with changes in the concentrations of specific metabolites (Fig. 2) and with changes in dominance of various microbial taxa (Fig. 4 and 5A). Furthermore, we highlight significant associations between the *Populus* metabolome and microbiome between two host species (Fig. 3 and 5B), which is likely partially responsible for the effects of host species on the microbiome seen throughout the literature (12, 35–37).

Consistent with previous research, we found that *S. musiva* is present in *P. deltoides* and *P. trichocarpa* but causes canker infection only in the latter (12) (Fig. 1). Further, we found a high abundance of *S. musiva* in the roots of plants, without noticeable symptoms of disease. *S. musiva* has been shown to overwinter in infected leaf litter. In the spring, ascospore production peaks, potentially leading to infection of new poplar leaves and shoots (38). Our results highlight the possibility that *S. musiva* may colonize roots from infected leaf litter. We hypothesize that like other microorganisms, *S. musiva* may be able to translocate from roots to leaves via plant transport vessels (39, 40). Further research is necessary to validate this hypothesis. Interestingly, our hypothesis that *S. musiva* load throughout the plant would be related to stem canker infection within *P. trichocarpa* or even within susceptible *P. trichocarpa* genotypes was unsupported by the data (Fig. 1) (12). *S. musiva* abundance can be used as a method of early disease detection before canker infections emerge (41). However, our results demonstrate that this method of detection is limited to the site of infection.

Our hypothesis that *S. musiva* abundance and stem canker infection would impact the *Populus* metabolome was marginally supported by the data (Fig. 2; Fig. S1). While we found strong differences in the leaf and root metabolome between *Populus* species (Fig. S1), as reported previously (19), differences in metabolome within species, specifically between infected and noninfected *P. trichocarpa* trees, were smaller and occurred only in the leaf. This is interesting because *S. musiva* also causes leaf spot on *Populus* leaves in addition to stem canker formation. While leaves were not scored for leaf spot at the time of collection, it is possible that changes in the leaf metabolome with stem canker infection occurred because of simultaneous leaf infection, although *S. musiva* leaf spot and canker susceptibility are not always correlated (38).

*In vivo* laboratory inoculations of *P. trichocarpa* with *S. musiva* have shown that *S. musiva* infection results in pronounced increases in defense compounds, such as salicylic acid and gentisic acid (i.e., 2,5-dihydroxybenzoic acid) in the stem (18). In contrast, we found that two phenolic glycosides, salirepin and salireposide, which are commonly associated with plant defense (29), were significantly enriched in uninfected *P. trichocarpa* leaves compared to their infected counterparts (Fig. 2). These phenolic glycosides are characteristic of members of the Salicaceae that include *Populus* sp. and *Salix* sp. Their functions in plant tissues are varied and can include being major components of the constitutive defense against pathogens and herbivores (42–44) as well as being a major increasing fraction of the osmotic constitution of leaves during acute drought (20) and being involved in attenuating incoming UV radiation in leaves exposed to high solar radiation (45). Given the bidirectional nature of pathogen establishment and plant defense, enrichment of these metabolites in uninfected trees could be responsible for reduced infection. However, future laboratory assays are necessary to definitively determine relationships between *Populus* metabolites and *S. musiva*.

Contrary to our hypothesis, across plant-associated habitats, the overall microbiome was robust to canker infection (Table 1). This is surprising considering that tree health is negatively impacted by canker infection, which should alter the flow of C, nutrients, and secondary metabolites throughout the plant, subsequently affecting microbial communities (46). In citrus, for instance, huanglongbing disease, associated with the bacterium “*Candidatus* Liberibacter asiaticus,” results in microbial disturbances throughout the plant (47). Instead, we found that the impacts of *S. musiva* on the microbiome were confined to certain microbial taxa and depended on local *S. musiva* abundance, not the formation of a stem canker infection. This suggests that the impact of *S. musiva* on the microbiome is localized and not the result of a systemic plant response. The ability of a single microbial member to impact the plant microbial community at large is generally a common trait of pathogenic species (48). However, the presence or high abundance of this pathogen did not always negatively affect the microbiome. Indeed, in some cases, *S. musiva* abundance was associated with greater microbial α-diversity (Fig. 3), and certain microbial phyla were positively associated with *S. musiva* abundance (Fig. 5). Hence, these results show that *S. musiva* may impact the plant microbiome even when a phenotypic infection is absent.

Associations between the plant metabolome and microbiome are well characterized (46), and our data somewhat concurs. This was most often the case in the leaf endosphere, where multiple metabolites were correlated with bacterial and archaeal α-diversity (Fig. 4) and the overall leaf metabolome was correlated with the overall leaf microbiome. Interestingly, two phenylpropanoid compounds, trans-3-*O*-caffeoylquinic acid and syringin, were positively correlated with archaeal and bacterial α-diversity in the leaf endosphere (Fig. 4). As phenylpropanoids function as defense compounds (49), this association could be indicative of antagonistic pressure by the plant on dominant pathogen members of the microbial community, creating niche space for rare, less-competitive microbial members. Alternatively, gentisic acid (2,5-dihydroxybenzoic acid) and azelaic acid, two other compounds important in plant defense of microbial pathogens (50), were negatively correlated with archaeal and bacterial α-diversity in the leaf endosphere (Fig. 4). It is possible that these defense compounds resulted in nonspecific impacts on the microbial community that negatively affected multiple microbial members. Lack of correspondence between the root metabolome and microbiome was somewhat surprising, because such associations have been shown in maize (51) and citrus (52). However, the core plant microbiome is often consistent among disparate species and even among different plant genera and families (53), suggesting that many microbial taxa found in endospheric habitats are adapted to this niche regardless of the chemical environment within the plant. This finding provides another line of evidence that other assembly factors besides selection, such as dispersal limitation (36) and more specifically priority effects (54), may be prominent in structuring the plant microbiome. Nevertheless, given the differences in the leaf metabolome and microbiome between *P. deltoides* and *P. trichocarpa*, it is likely that the leaf microbiome is somewhat mechanistically related to its chemical environment.

Since the leaf metabolome and microbiome were related, *S. musiva* may impact the leaf microbiome indirectly through its effect on leaf metabolites (Fig. 2). Indeed, stearic acid, which was enriched in the leaves of *P. trichocarpa* infected with *S. musiva* stem cankers, was correlated with the relative abundance of multiple microbial genera (Fig. 5C). Accumulation of fatty acids, including stearic acid, is associated with greater chloroplast abundance and energy production (55, 56). Interestingly, these fatty acids can be fungus-derived metabolites that are higher with increased fungal abundance. In this study, we cannot determine whether fatty acids are plant or fungus derived; thus, future studies should employ integration of host and microbiome transcriptomics and proteomics to tease out the source of these metabolites (19). Regardless of origin, increases in fatty acid accumulation are likely key for plant-microbe signaling (57) and have been shown to play a role in host-pathogen communication and pathogen colonization, modulating the ability of pathogenic microbes, such as *Xanthomonas*, *Cladosporium*, *Didymella*, and *Ramularia*, to become established (Fig. 5C) while being negatively associated with other plant pathogens, such as *Alternaria* and *Venturia.* This highlights the complexity of metabolome-microbiome interactions but establishes a nascent systems understanding of how *S. musiva* might impact the *Populus* holobiont.

By taking a systems-based approach, we show that the effects of *S. musiva* on the *Populus* holobiont occur outside the site of infection (i.e., the stem canker). Our research contributes to improving the fundamental understanding of *S. musiva* and *Populus* sp. ecology and the utility of a holobiont approach in understanding plant disease. However, further experimental work is necessary to mechanistically corroborate relationships between *S. musiva*, the *Populus* metabolome, and *Populus* microbiome observed in this study. Additionally, as recent work has shown that the *Populus* microbiome is temporally variable (33), these results will need to be validated across seasons.

## MATERIALS AND METHODS

### Infection data and sample collection.

On 26 August 2019, roots and the attached soil were collected from 75 2-year-old *Populus* trees consisting of 10 genotypes and two species (*P. deltoides* and *P. trichocarpa*) from the Blount County Common Garden (see reference 36 for site and genotype collection details). Of the eight *P. trichocarpa* genotypes, half were characterized as resistant to *S. musiva* and half as susceptible to *S. musiva* based on prior greenhouse inoculation trials (16). In prior inoculation trials, these resistant genotypes showed some degree of tolerance to *S. musiva* infection (16).

Leaf samples were collected from the canopy. About four leaves were immediately frozen on dry ice for metabolite analysis, and about four leaves were placed on blue ice (4°C) for microbiome processing and were processed within three days. Leaves for microbiome analysis were rinsed (rinsate was collected as leaf surface samples) and surface sterilized as previously described (36) by washing leaves with bleach and rinsing the leaves with autoclaved water (four times). Root samples were excavated by use of hand tools and tracing large roots connected to the primary stem. Root samples were divided into groups—one for metabolomic analysis and one for microbiome analysis. Roots for metabolomic analysis were shaken to remove excess soil and were immediately placed on dry ice. Roots for microbiome analysis were immediately placed on dry ice with attached soil. Roots for microbiome analysis, and the attached soil (operationally defined as rhizosphere soil), were stored at −80°C until root washing with sterile water (the rinsate of which was collected as the rhizosphere fraction), sterilization, and genomic DNA (gDNA) extractions took place (12). Briefly, fine roots (<2-mm diameter) were sorted and surface sterilized by sequential washing with bleach (3.125%) and then ethanol (70%) and rinsing the roots with autoclaved water (four times) as previously described (12), and the rhizosphere was collected from an initial rinse with sterile water. Sterility of the root surface was validated by streaking water from the final rinse across an R2A agar plate and incubating for 48 h at 20°C to check for the appearance of colonies, as previously described (12). Samples with colonies present had this sterilization procedure repeated.

During the dormant period before leaf out on 9 March 2020, all trees were inventoried for *S. musiva* canker infection. Briefly, the overall number of main stem cankers was determined, and their severity was visually scored on a scale of 1 to 5, where 1 is equivalent to the discoloration and slight depression associated with early colonization and 5 indicates severe cankers causing stem breakage (38). Trees with any symptom of canker infection were subsequently categorized as infected.

### DNA extractions, PCR amplification, sequencing, and bioinformatics.

Prior to extraction, washed leaf and root tissues were cut into fine pieces (~5 mm or less), leaf and root rinsates (leaf surface and rhizosphere samples, respectively) were centrifuged at 10,000 × *g*, and the supernatant was removed. These pelleted leaf surface and rhizosphere samples were then extracted using the Qiagen PowerSoil DNA kit (Germantown, MD, USA) following the standard protocol except that a Precellys tissue homogenizer (Bertin Technologies, Montigny-le-Bretonneux, France) was used to bead beat extractions (30 s of 5,500 × *g* bead beating with a 30-s rest, in triplicate). Root and leaf samples were extracted using the Qiagen PowerPlant Pro DNA kit (Qiagen, Germantown, MD, USA) following the standard protocol except that prior to extraction, 50 mg of tissue per extraction was bead-beaten for 1 min in liquid nitrogen blocks with one sterile steel bead twice. We used a Zymo DNA Clean and Concentrator-5 kit (Zymo Research Corporation, Irvine, CA, USA) to purify and concentrate plant tissue extractions prior to PCR amplification. Extractions were quantified using the Qubit dsDNA BR assay kit (Invitrogen, Waltham, MA).

A two-step PCR approach was used with barcode-tagged templates and primers targeting the V4 region of the 16S rRNA gene for *Archaea* and *Bacteria* and the ITS2 region for *Fungi* using pooled primer sets to increase coverage of archaeal, bacterial, and fungal taxa (Table S2). The first step of PCR included 2.5 μM peptide nucleotide acid (PNA) blockers for 16S rRNA amplifications (GGCAAGTCTTCTTCGGA and GGCTCAACCCTGGACAG), and 2.5 μM concentrations of PNA targeting plant nuclear rRNA genes for the ITS2 region (CGAGGGCACGTCTGCCTGG) were used to reduce amplification of plant material. Each reaction mixture contained 2 μL of template DNA, a 0.25 μM concentrations of the primer pair, 1× KAPA HiFi HotStart ReadyMix, and molecular-grade water for a total reaction volume of 25 μL. PCR amplifications were performed with the conditions 95°C for 3 min, 25 cycles (30 cycles for endosphere) of 95°C for 30 s, 78°C for 30 s, 55°C for 30 s and 72°C for 30 s and a final extension of 72°C for 5 min. The second step of PCRs was amplification following the Illumina 16S metagenomic sequencing library preparation instructions with the conditions 95°C for 3 min, 8 cycles of 95°C for 30 s, 55°C for 30 s, and 72°C for 30 s, and a final extension of 72°C for 5 min.

10.1128/msystems.00120-22.2 TABLE S2 Primer (red) and Illumina adapter (black) sequences for PCR amplification. Download Table S2, DOCX file, 0.01 MB. Copyright © 2022 Dove et al. 2022 Dove et al. https://creativecommons.org/licenses/by/4.0/ This is an open-access article distributed under the terms of the Creative Commons Attribution 4.0 International license.

After PCRs, all experimental units were pooled based on band intensity and purified with Agencourt AMPure XP beads (0.7:1 bead-to-DNA ratio; Beckman Coulter Inc., Pasadena, CA, USA). Paired end sequencing (2 × 251) was completed on pooled prepared libraries on an Illumina MiSeq instrument (Illumina, San Diego, CA) at Oak Ridge National Laboratory using V2 chemistry and included a ≥15% PhiX sequencing control library.

Both 16S and ITS2 data sets were denoised, joined, delineated into amplicon sequence variants (ASVs), and assigned taxonomy in the QIIME2 environment (v. 2019.7) (58). Prior to ASV delineation using DADA2 (59), 16S reads were truncated to 200 bases (to remove low-quality base calls), with the first 19 bases trimmed (to remove primers). For ITS2, reads were trimmed (including primers) using the ITSxpress plugin under the default parameters (60) with no further trimming/truncation prior to ASV delineation. We then assigned representative sequences a taxonomic classification using the naive Bayes classifier through the sklearn Python package for 16S rRNA sequences with the SILVA database (release 132) (61) and a confidence of 0.7. We assigned taxonomic classifications of ITS2 of the ribosomal operon to representative sequences using consensus BLAST (identity, 80%; E value, 0.001; minimum fraction of assignments, 0.51) (62) and the UNITE reference database (version 8.0) (63). We removed 16S reads assigned as mitochondria and chloroplasts and kept only reads assigned to *Bacteria* and *Archaea* (~65% of reads were retained). All ITS reads were assigned to the fungal kingdom.

### *S. musiva* abundance.

*S. musiva* abundance was measured by quantitative PCR (qPCR) targeting the beta-tubulin gene as described by Abraham et al. (41) (NABtF: 5′-CGACCTGAACCACCTTGTCT-3′ and NABtR: 5′-CACGGTAACAGCGCGGAACGA-3′). Template DNA concentrations were normalized to 10 ng μL^−1^, and PCRs were conducted in a 384-well plate containing 1× SYBR green (iTaq Universal SYBR Green Supermix), 500 nmol of each primer, and 2 μL of template DNA for a total volume of 20 μL. To generate a standard curve, *S. musiva* DNA standards were extracted from a cultured representative (*S. musiva* MN14), diluted 1:10, 1:100, 1:1,000 and 1:10,000, and quantified using the Qubit dsDNA BR assay kit (Invitrogen, Waltham, MA). To detect nonspecific amplification, negative controls (no-template control and Marssonina brunnea 441) were included in the assays. The reactions were carried out using a 7900HT fast real-time PCR machine (Applied Biosystems, Waltham, MA, USA) under the following conditions: initial denaturation at 95°C for 10 min, followed by 40 cycles of amplification at 95°C for 15 s and 58°C for 30 s.

### Metabolite extraction and processing.

Leaf and root tissue metabolites were processed and analyzed by gas chromatography-mass spectrometry (GC-MS). Tissues stored at −80°C were lyophilized and then powdered using a Spex Geno-Grinder (Metuchen, NJ, USA). Approximately 25 mg of powdered leaf and 45 mg of powdered root material was extracted twice with 80% ethanol. Sorbitol (75 μL; 1 mg mL^−1^) was added to the first extract and used as an internal standard. After the extracts were combined, a 1-mL aliquot of the leaf extract and a 250-μL aliquot of the root extract were dried under a stream of nitrogen. Both types of samples were then derivatized by dissolving aliquots in 500 μL acetonitrile followed by addition of 500 μL of *N*-methyl-*N*-(trimethylsilyl)trifluoroacetamide with 1% trimethylchlorosilane (MSTFA + 1% TMCS) and heating at 70°C for 1 h to produce trimethylsilyl (TMS) metabolite derivatives. After 2 days, 1 μL was injected into the GC-MS and analyzed using GC-MS parameters described previously (38). Metabolites were identified using the Wiley Registry (10th edition) as well as a large, user-created database of TMS-derivatized metabolites. Metabolites were quantified relative to the internal standard and normalized to the mass extracted, extract volume analyzed, and injection volume.

### Statistical analyses.

All statistical analyses were conducted in R v. 4.0.2 (64) with the hillR (65), phyloseq (66), and vegan (67) packages. For all statistical tests, significance was defined at the level of *P* values of 0.05. The R code used to conduct statistical analyses and generate figures can be found at https://github.com/nicholascdove/S_musiva_project.

Differences in log-transformed *S. musiva* abundance among species, genotypes, or *S. musiva* resistance and level of infection were assessed by two-way ANOVA for each sample type. Where independent variables were significant, we assessed multiple comparisons by Tukey’s honestly significant difference (HSD) test (this was done for subsequent ANOVA as well). We used Q-Q plots and scale-location plots to inspect normality and homoscedasticity, respectively (this was done for subsequent ANOVA as well).

Differences in metabolites among species, genotypes, *S. musiva* resistance, and *S. musiva* canker infection were first assessed by PERMANOVA (using Euclidean distances) to determine if the overall metabolome differed. Tests including the latter three factors included only *P. trichocarpa* genotypes, because only these trees had differential *S. musiva* resistance and *S. musiva* canker infection (i.e., *P. deltoides* did not show canker symptoms). This was visualized using principal-component analysis. Wilcoxon rank sum tests followed by a false discovery rate (FDR) *P* value adjustment were used to determine differentially abundant metabolites between infected and noninfected *P. trichocarpa* trees. Spearman rank sum tests were used to determine correlations between metabolites and *S. musiva* abundance, and *P* values were corrected by the FDR.

Differences in α-diversity were compared by means of Hill numbers (68) of samples rarefied to 200 reads for 16S leaf endospheres, 3,000 reads for 16S leaf surfaces, 8,000 reads for 16S root endospheres, 10,000 reads for 16S rhizospheres, 2,000 reads for ITS leaf endospheres, 4,000 reads for ITS leaf surfaces, 3,000 reads for ITS root endospheres, and 20,000 reads for ITS rhizospheres (the average integer of reads was used after 999 rarefactions) at orders of *q* of 0, 1, and 2 (rarefaction curves are presented in Fig. S3). Hill numbers express the effective diversity of a sample (i.e., the number of equally abundant species that would be needed to give the same value of a diversity measure) among different metrics of *q* (33). Because the parameter *q* determines the relative weighting of rare species, multiple traditional α-diversity indices (e.g., richness, Shannon’s diversity, and Simpson’s diversity) can be compared in a unified framework by adjusting the *q* metric. For instance, at a *q* of 0, all species are weighted equally (richness); at a *q* of 1, species are weighted proportionally to their relative abundance (analogous to Shannon’s index); and at a *q* of 2, rare species are down-weighted (analogous to Simpson’s index). Differences in means of Hill numbers between host species, *S. musiva* resistance, and *S. musiva* canker infection were assessed by Wilcoxon rank sum tests for each sample type and orders of *q*. Tests involving *S. musiva* resistance and *S. musiva* canker infection included only *P. trichocarpa* genotypes, because only these trees had differential *S. musiva* resistance and *S. musiva* canker infection (i.e., *P. deltoides* did not show signs of canker infection). Spearman rank sum tests were used to determine correlations between Hill numbers and *S. musiva* abundance for each sample type and order of *q*. This was also done to determine correlations between Hill numbers and plant metabolites, and *P* values were corrected by the FDR.

10.1128/msystems.00120-22.5 FIG S3 Rarefaction curves across amplicons, sample types, and *S. musiva* resistance. Dotted lines represent minimum read depth and rarefaction level for α-diversity analyses for each amplicon-sample type combination (root endosphere 16S, 8,000; rhizosphere 16S, 10,000; root endosphere ITS, 3,000; rhizosphere ITS, 20,000). Axes vary among plots. Download FIG S3, JPG file, 0.7 MB. Copyright © 2022 Dove et al. 2022 Dove et al. https://creativecommons.org/licenses/by/4.0/ This is an open-access article distributed under the terms of the Creative Commons Attribution 4.0 International license.

Differences in the community composition of the archaeal and bacterial and the fungal microbiomes among species, *S. musiva* abundance, genotypes, *S. musiva* resistance, and *S. musiva* canker infection were assessed by PERMANOVA (69) for each sample type. Tests including the latter three factors included only *P. trichocarpa* genotypes, because only these trees had differential *S. musiva* resistance and *S. musiva* canker infection (i.e., *P. deltoides* did not show signs of canker infection). For the PERMANOVA, we used Bray-Curtis dissimilarity applied to proportionally normalized data (i.e., not rarefied). Relationships between *S. musiva* abundance and the microbiome were further investigated by ANCOM-BC (70). Relationships between the microbiome and metabolome were explored using Mantel tests and were further investigated using ANCOM-BC.

### Data availability.

Metabolomic and qPCR data as well as data for the scoring of infected *Populus* are archived in the Dryad repository (https://doi.org/10.6071/M3CM2F). All sequence data can be accessed through the sequence read archive under BioProject no. PRJNA804020.
