# Supplementary figures and images for: Relationships between Sphaerulina musiva Infection and the Populus Microbiome and Metabolome
Source: mSystems. 2022 Jul 18;7(4):e00120-22. doi: 10.1128/msystems.00120-22 (PMC9426494; doi:10.1128/msystems.00120-22)

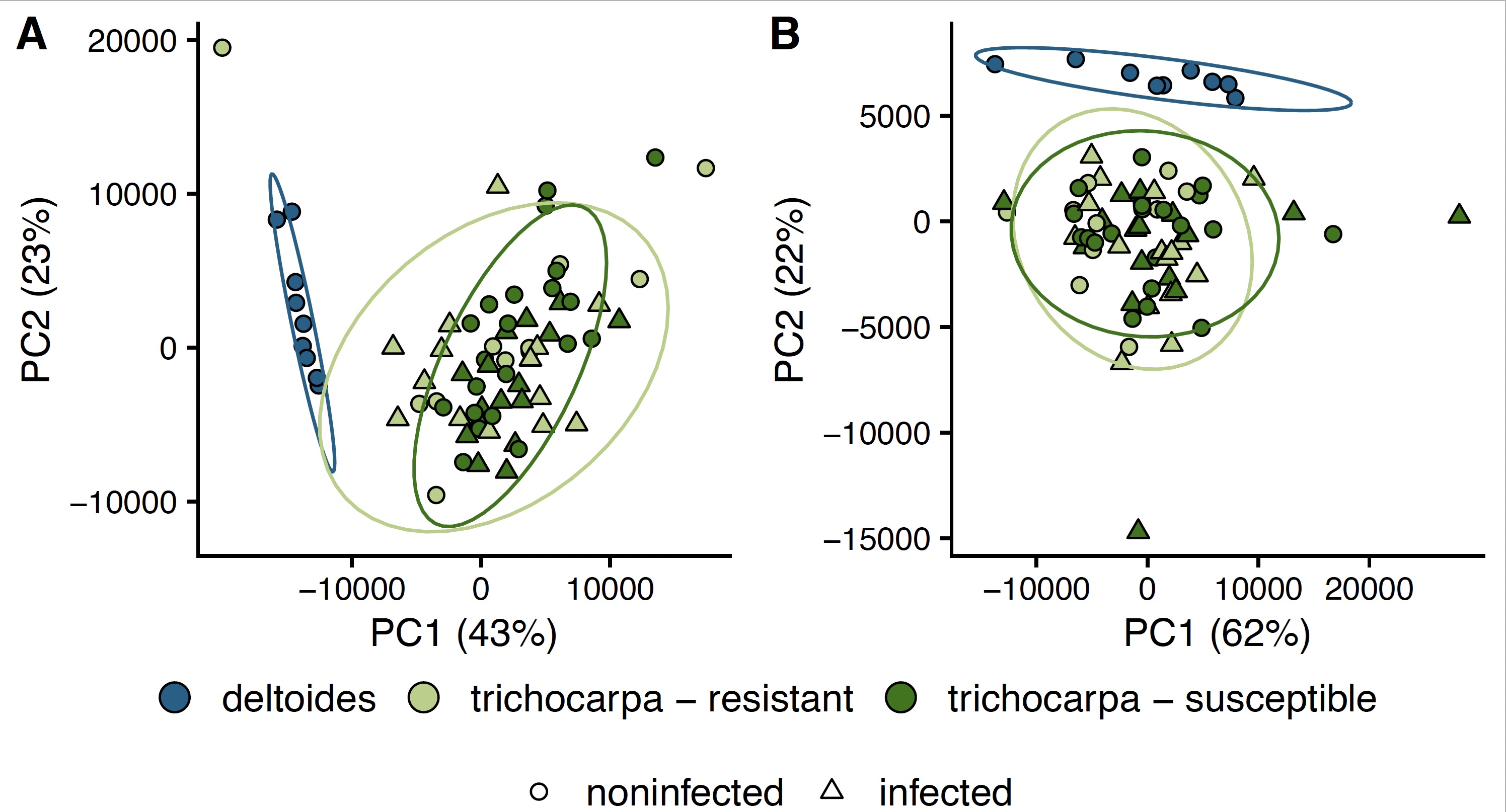

Supplement: FIG S1 [file msystems.00120-22-s0003.jpg]

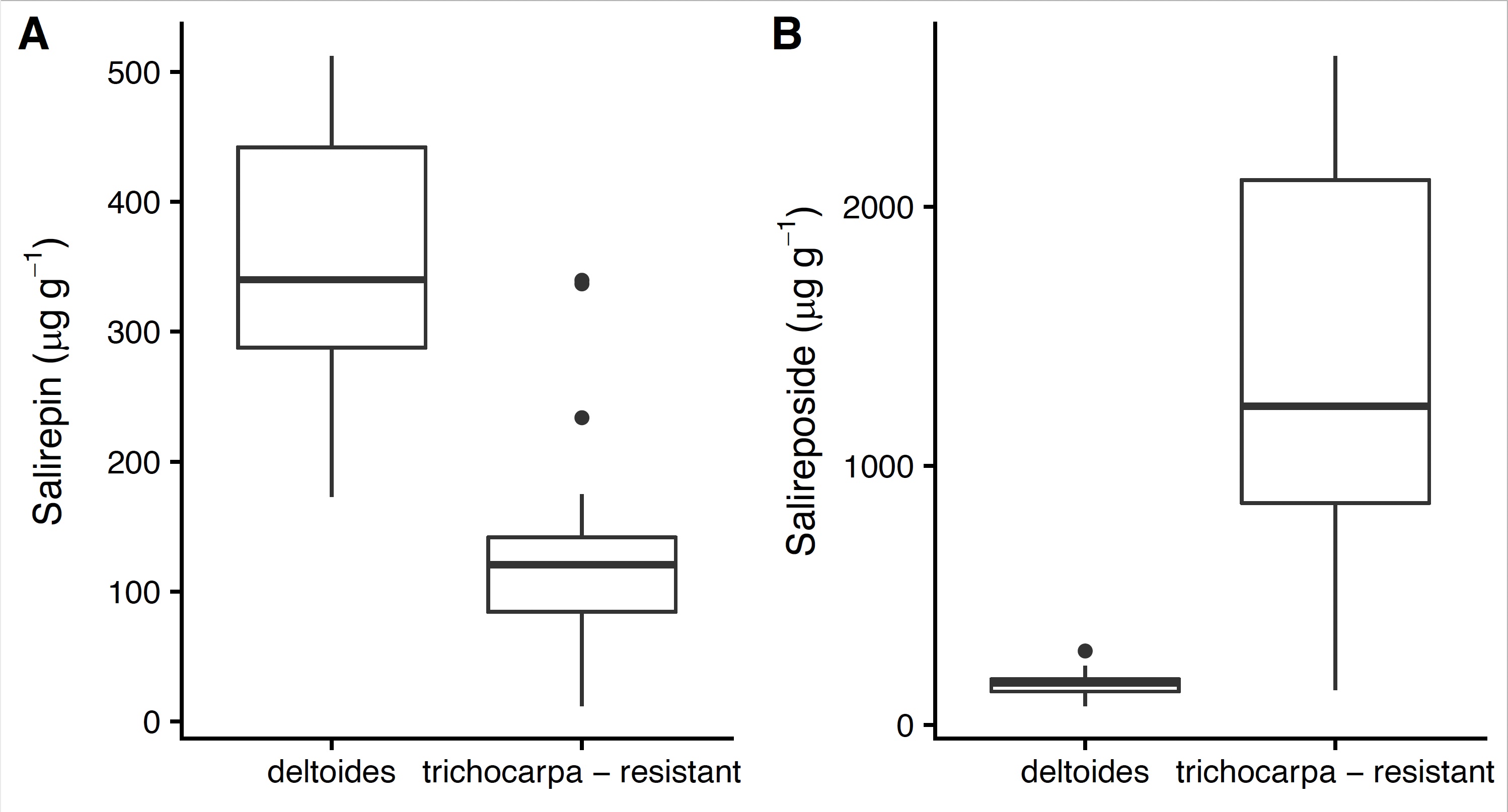

Supplement: FIG S2 [file msystems.00120-22-s0004.jpg]

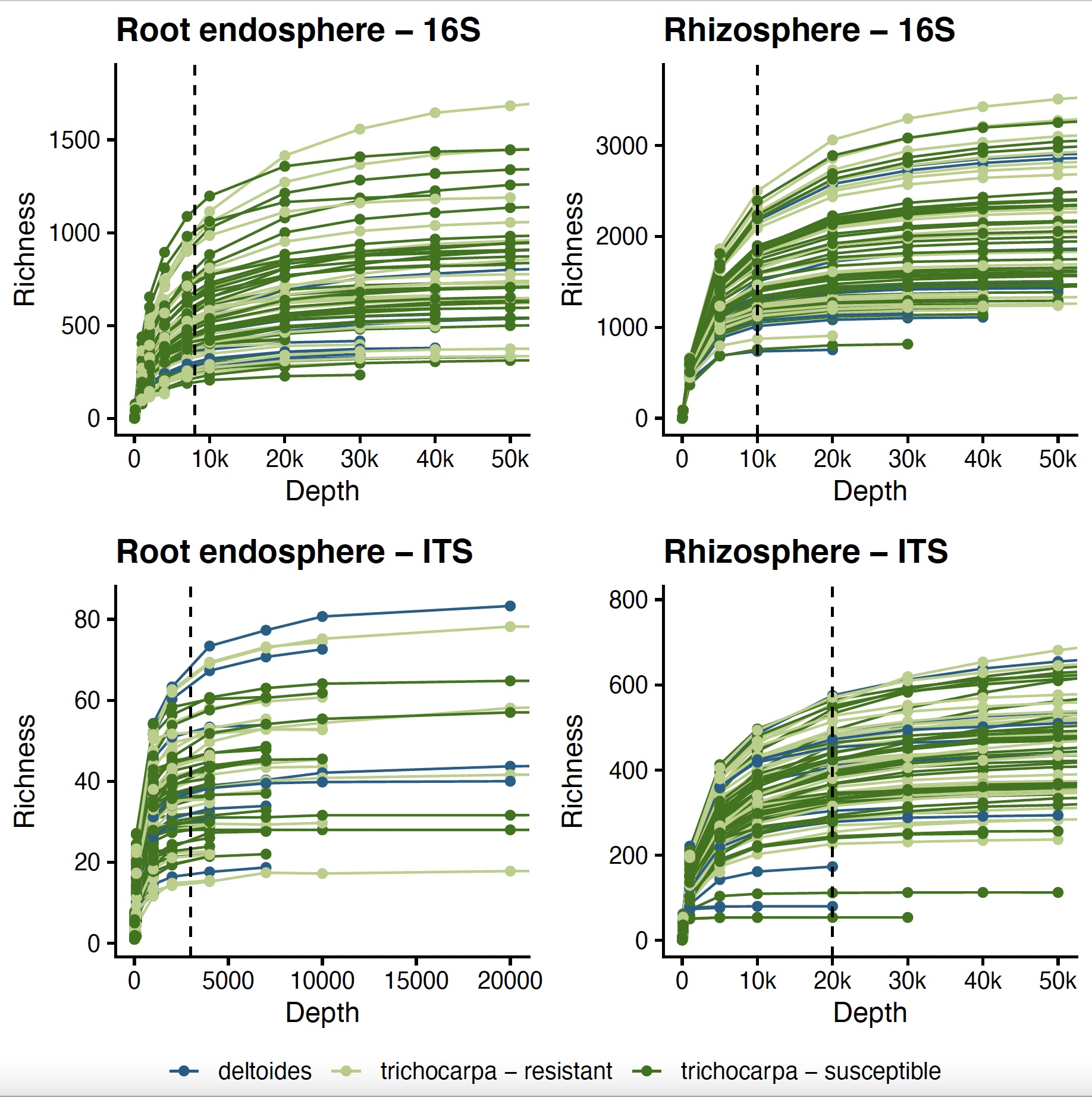

Supplement: FIG S3 [file msystems.00120-22-s0005.jpg]
