# Supplementary material for: Relationships between Sphaerulina musiva Infection and the Populus Microbiome and Metabolome
Source: mSystems. 2022 Jul 18;7(4):e00120-22. doi: 10.1128/msystems.00120-22 (PMC9426494; doi:10.1128/msystems.00120-22)
Supplement: TABLE S1 [file msystems.00120-22-s0001.docx]

**Table S1**

|  |  |  |  | *P. trichocarpa* only | |
| --- | --- | --- | --- | --- | --- |
| Amplicon | Sample type | q | Species | Resistance | Infection |
| 16S | Leaf Endosphere | 0 | **0.002** | 0.354 | 0.897 |
|  |  | 1 | **0.002** | 0.719 | 0.562 |
|  |  | 2 | **0.002** | 0.909 | 0.331 |
|  | Leaf Surface | 0 | 0.473 | 0.061 | 0.358 |
|  |  | 1 | 0.758 | 0.057 | 0.662 |
|  |  | 2 | 0.520 | 0.092 | 0.972 |
|  | Root Endosphere | 0 | **0.039** | 0.898 | 0.446 |
|  |  | 1 | **0.010** | 0.554 | 0.513 |
|  |  | 2 | 0.066 | 0.585 | 0.485 |
|  | Rhizosphere | 0 | 0.422 | 0.966 | 0.955 |
|  |  | 1 | 0.802 | 0.966 | 0.655 |
|  |  | 2 | 0.250 | 0.977 | 0.845 |
| ITS | Leaf Endosphere | 0 | 0.099 | 0.408 | 0.527 |
|  |  | 1 | 0.592 | 0.447 | 0.790 |
|  |  | 2 | 0.637 | 0.133 | 0.738 |
|  | Leaf Surface | 0 | **0.014** | 0.214 | 0.123 |
|  |  | 1 | 0.150 | 0.096 | 0.318 |
|  |  | 2 | 0.391 | 0.142 | 0.453 |
|  | Root Endosphere | 0 | 0.386 | 0.376 | 0.231 |
|  |  | 1 | 0.812 | 0.725 | 0.466 |
|  |  | 2 | 0.628 | 0.686 | 0.744 |
|  | Rhizosphere | 0 | 0.959 | 0.223 | 0.955 |
|  |  | 1 | **0.033** | 0.113 | 0.511 |
|  |  | 2 | **0.036** | 0.129 | 0.502 |
