# Supplementary material for: Relationships between Sphaerulina musiva Infection and the Populus Microbiome and Metabolome
Source: mSystems. 2022 Jul 18;7(4):e00120-22. doi: 10.1128/msystems.00120-22 (PMC9426494; doi:10.1128/msystems.00120-22)
Supplement: TABLE S2 [file msystems.00120-22-s0002.docx]

**Table S2**

| **PRIMER NAME** | **SEQUENCE** | **DIRECTION** | **TARGET** | **REFERENCE** |
| --- | --- | --- | --- | --- |
| ITS3NGS1 | TCGTCGGCAGCGTCAGATGTGTATAAGAGACAGCATCGATGAAGAACGCAG | Forward | Fungi | White *et al.* 1990^1^ |
| ITS3NGS2 | TCGTCGGCAGCGTCAGATGTGTATAAGAGACAGCAACGATGAAGAACGCAG | Forward | Chytridiomycota | Tedersoo *et al.* 2014^2^ |
| ITS3NGS3 | TCGTCGGCAGCGTCAGATGTGTATAAGAGACAGCACCGATGAAGAACGCAG | Forward | Sebacinales | Tedersoo *et al.* 2014^2^ |
| ITS3NGS4 | TCGTCGGCAGCGTCAGATGTGTATAAGAGACAGCATCGATGAAGAACGTAG | Forward | Glomeromycota | Tedersoo *et al.* 2014^2^ |
| ITS3NGS5 | TCGTCGGCAGCGTCAGATGTGTATAAGAGACAGCATCGATGAAGAACGTGG | Forward | Sordariales | Tedersoo *et al.* 2014^2^ |
| ITS3NGS10 | TCGTCGGCAGCGTCAGATGTGTATAAGAGACAGCATCGATGAAGAACGCTG | Forward | Stramenopila | Tedersoo *et al.* 2014^2^ |
| ITS4NGR | GTCTCGTGGGCTCGGAGATGTGTATAAGAGACAGTCCTSCGCTTATTGATATGC | Reverse | Fungi | White *et al.* 1990^1^ |
| ARCH-ITS4 | GTCTCGTGGGCTCGGAGATGTGTATAAGAGACAGTCCTCGCCTTATTGATATGC | Reverse | Archaearhizomycetes | Cregger *et al.* 2018^3^ |
| 515F | TCGTCGGCAGCGTCAGATGTGTATAAGAGACAGGTGCCAGCMGCCGCGGTAA | Forward | Bacteria/Archaea | Lane *et al.* 1985^4^ |
| 515F_f1C | TCGTCGGCAGCGTCAGATGTGTATAAGAGACAGGTGCCAGCMGCWGCGGTAA | Forward | Cloroflexi | Shakya *et al.* 2013^5^ |
| 515F_f1TM7 | TCGTCGGCAGCGTCAGATGTGTATAAGAGACAGGTGCCAGCMGCCGCGGTCA | Forward | TM7 | Shakya *et al.* 2013^5^ |
| 515F_f4Arc | TCGTCGGCAGCGTCAGATGTGTATAAGAGACAGGTGKCAGCMGCCGCGGTAA | Forward | Archaea | Shakya *et al.* 2013^5^ |
| 806R | GTCTCGTGGGCTCGGAGATGTGTATAAGAGACAGGGACTACHVGGGTWTCTAAT | Reverse | Bacteria/Archaea | Lane *et al.* 1985^4^ |

^1^ White TJ, Bruns T, Lee S, Taylor J. 1990. Amplification and direct sequencing of fungal ribosomal RNA genes for phylogenetics. In: Innis MA, Gelfand DH, Sninsky JJ, White TJ (eds) PCR Protocols: a Guide to Methods and Applications. New York: Academic Press, 315-322.

^2^ Tedersoo L, Bahram M, Põlme S, Kõljalg U, Yorou NS, Wijesundera R, Ruiz LV, Vasco-Palacios AM, Thu PQ, Suija A, *et al.* 2014. Global diversity and geography of soil fungi. *Science* 346: 1256688.

^3^ Cregger MA, Veach AM, Yang ZK, Crouch MJ, Vilgalys R, Tuskan GA, Schadt CW. 2018. The Populus holobiont: dissecting the effects of plant niches and genotype on the microbiome. *Microbiome* 6: 31.

^4^ Lane DJ, Pace B, Olsen GJ, Stahl DA, Sogin ML, Pace NR. 1985. Rapid determination of 16S ribosomal RNA sequences for phylogenetic analyses. *Proceedings of the National Academy of Sciences* 82: 6955–6959.

^5^ Shakya M, Gottel N, Castro H, Yang ZK, Gunter L, Labbé J, Muchero W, Bonito G, Vilgalys R, Tuskan G, *et al.* 2013. A multifactor analysis of fungal and bacterial community structure in the root microbiome of mature *Populus deltoides* trees. *PLoS ONE* 8: e76382
